# Origins of Negative Differential Resistance in N-doped ZnO Nano-ribbons: Ab-initio Investigation

**DOI:** 10.1038/s41598-019-46335-0

**Published:** 2019-07-09

**Authors:** Alaa Shaheen, Muhammad Ali, Wael Othman, Nacir Tit

**Affiliations:** 1 Physics Department, College of Science, UAE University, P. O. Box 15551, Al-Ain, United Arab Emirates; 2 Masdar Institute, Khalifa University of Science and Technology, Abu Dhabi, United Arab Emirates; 3 School of Materials Science and Engineering, Zhejiang University, Hangzhou, 310027 P. R. China

**Keywords:** Electronic devices, Electronic properties and materials

## Abstract

The electronic transport in low-dimensional materials is controlled by quantum coherence and non-equilibrium statistics. The scope of the present investigation is to search for the origins of negative-differential resistance (NDR) behavior in N-doped ultra-narrow zigzag-edge ZnO nano-ribbons (ZnO-NRs). A state-of-the-art technique, based on a combination of density-functional theory (DFT) and non-equilibrium Green’s function (NEGF) formalism, is employed to probe the electronic and transport properties. The effect of location of N dopant, with respect to the NR edges, on IV-curve and NDR is tested and three different positions for N-atom are considered: (i) at the oxygen-rich edge; (ii) at the center; and (iii) at the Zn-rich edge. The results show that both resistance and top-to-valley current ratio (TVCR) reduce when N-atom is displaced from O-rich edge to center to Zn-rich edge, respectively. After an analysis based on the calculations of transmission coefficient versus bias, band structures, and charge-density plots of HOMO/LUMO states, one is able to draw a conclusion about the origins of NDR. The unpaired electron of N dopant is causing the curdling/localization of wave-function, which in turn causes strong back-scattering and suppression of conductive channels. These effects manifest themselves in the drawback of electric current (or so called NDR). The relevance of NDR for applications in nano-electronic devices (e.g., switches, rectifiers, amplifiers, gas sensing) is further discussed.

## Introduction

Although research on zinc oxide (ZnO) commenced as early as 1935^1^, earlier than research on many semiconductors, only the recent few decades have witnessed several breakthroughs paving the way for revolutionary applications that exploit the multifunctional properties of ZnO and making it highly competitive in the market^2–4^. Amongst the breakthroughs worth mentioning are: (i) the discovery of p-type doping in ZnO using (Li,N)^5,6^ which enhanced the applications of ZnO in electronics; and (ii) the invention of one-atomic thick graphene by Novoselov and Geim^7^ which opened the opportunity for many other materials to get synthesized into low-dimensional nanostructures (e.g., ZnO, BN, MoS_2_, silicone, germanene, black-phosphorus, etc.)^8–10^. From the perspectives of native properties, ZnO crystallizes into wurtzite structure with a high direct band-gap energy of 3.3 eV and an outstanding excitonic binding energy of 60 meV at room temperature. Adding to its low fabrication cost, it becomes not only more competitive than GaN in optoelectronics but rather comprises much more fields of applications; (iii) the advent in modern growth techniques ranging up to the state-of-the-art flow-rate modulated molecular beam epitaxy (MBE), using which numerous structures have been realized and have long seemed prone to be embryonic^11,12^.

By nature, ZnO has the tendency to grow into nanorods. Electrons in these systems are free to move in one dimension, but their restricted motion in the other two directions is governed by quantum mechanics, especially when the cross-sectional size of nanorod is at nanometer scale. As a matter of fact, nanorods possess special characteristics completely different from the bulk ones^13^. Such properties make them suitable for even broader range of applications for instance in: (a) nano-electronics: ZnO nanorods are utilized as extended gate in MOSFET^14^, and logical circuits^15^; (b) nano-photonics: ZnO nanorod arrays were used as photonic crystals^16^, as light waveguide^17^, as LED^18^, as radiation detector^19^ and in dye-sensitized solar cells^20^; (c) biomedicine: ZnO nanorods and nanoparticles are utilized in biological and biomedical application (diagnosis and therapy) as to possess high radiative efficiency and least toxicity^21,22^; (d) Spintronics: Magnetic studies showed that Co-doped ZnO nanorods exhibited room-temperature ferromagnetism with a magnetization increasing with Co content^23^; (e) Gas-sensing: ZnO besides SnO_2_ are among the leading materials in gas sensing applications. ZnO nanorods in particular have demonstrated recently very high sensitivity to detect H_2_S at room temperature with a selectivity at the order of ppb^24^; (f) Bio-sensing: detection of glucose was achieved using ZnO nanorod array^25^.

From structural point of view, it is worth to mention that nanorods are not the only quasi-one-dimensional nanostructures of ZnO. A review by Z.L. Wang elaborated that ZnO has likely the richest family of nanostructures both in structures and properties^26^. ZnO have been synthesized for instance in nanobelts^27^, nanowires^28^, tetrapods^29^, and nanoribbons^30,31^. Different structures are fabricated by different growth methods to yield diversity of interesting applications comprising optoelectronics, catalysis, piezoelectricity, sensing and nanoelectronics. The present investigation focuses on ZnO nanoribbons (ZnO-NR) and their applications in nano-electronics, especially as N-doping is found to yield negative differential resistance (NDR) behavior at low bias. Experimental evidence of NDR has recently been reported on ZnO-based nano-devices^32,33^. Because of its essential role and applications in high-frequency oscillators, frequency multipliers and logical gates^34^, the understanding of the origins of NDR (i.e., reasons behind its appearance and its mechanisms) do deserve full investigation and are the scope of the present paper for the case of ZnO-NR:N based devices.

Historically, the negative-differential resistance (NDR) and the quantum tunneling were simultaneously discovered by Esaki in 1958 and earned him Nobel Prize in Physics in 1973^35^. The discovery gave birth to new electronic devices starting from Esaki Diode (i.e., “tunnel diode”). Subsequently, J.B (Ian) Gunn further discovered the Gunn’s effect in 1962, when he observed random noise-like oscillations, validated by the existence of NDR, after applying a bias on n-type GaAs samples and crossing a certain threshold^36^.

A “Gunn diode” (i.e., named also transferred electron device “TED”) is a form of diode proven itself useful in high-frequency electronics. The “Gunn” diode is mainly composed of three regions: two of them are heavily n-doped in the sides of the two terminals, and a thin layer of lightly n-doped material in between, while conventional diodes are usually made of a homo-junction with n- and p-doped regions. “Gunn Diode” can be fabricated using semiconducting materials, such as GaAs, InP, and GaN, which possess direct bandgap with two valleys in conduction bands. Actually, the 2-valley model has been broadly accepted as a physical explanation to the Gunn effect. The model states that the current raises with the increasing applied voltage until certain energy threshold at which the upper CB valley starts to get filled. The upper CB valley (U-valley) has higher effective mass than the lower CB valley (L-Valley), so the current drops as a compromise between the simultaneous fillings for two CB valleys; i.e., J = e(µ_L_n_L_↓ + µ_U_n_U_↑), as the population of L-valley (of higher mobility) decreases and the population of U-valley (of lower mobility) increases. Within the regime of NDR, alternating current oscillations take place with frequency/period being dependent on the material’s gap/valleys and active region’s length. Gunn diodes are routinely used to fabricate oscillators in the 10-GHz and higher (THz) frequency range. For instance GaAs-based Gunn diodes are made with frequencies up to 200 GHz, whereas GaN-based ones can reach up to 3 THz^37^. Furthermore, both Esaki and Gunn diodes use NDR for applications as oscillators, amplifiers, frequency converters, and detectors^38,39^.

NDR manifests itself as a quantum mechanical phenomenon that can be observed in nano-electronic devices and is, as a matter of fact, governed by quantum coherence and statistical mechanics. Because of its importance in diversity of applications in nano-electronics, search for existence of NDR and its origins have attracted enormous interests. Until date, such behavior has been observed and predicted in molecular electronic devices^40–45^, and in functionalized graphene nano-ribbons^46–49^ and in carbon nanotubes (CNTs)^50^.

In computational side, a state-of-the-art technique based on a combination of the density-functional theory (DFT) and the non-equilibrium Green’s functions (NEGF) formalism has broadly been employed in attempting to explain the NDR behavior in nanoscale devices. The methods are incorporated into the code of Atomistic Tool-Kit (ATK)^51^. While debates about the origins and mechanisms yielding NDR are still active, the so-far achieved findings can be categorized into three possible groups: (i) Two-valley model has been successful to explain the Gunn effect as stated above^36–39^; (ii) Suppression of resonant band/channel at Fermi level which is the case of molecular junctions^40–45^. Using ATK, Fan and coworkers^52^ demonstrated that a single C_60_-molecule in a nanodevice can yield NDR controllable by shape deformation of molecule and specifically its interactions with the leads. Such weak interactions have the key control on states at Fermi level and can tune NDR behavior. Ling-Na and coworkers^53^ showed that the transport properties in molecular junctions can be modulated by doping. Namely, N-doping at shoulder of crossed graphene nano-ribbon (GNR) has ability to tune NDR behavior at low bias. Furthermore, the weak bonding of hydrogen at edges in GNR has ability to control the bands at Fermi level. It has been shown by Chauhan and coworkers^54^ that B-doping of GNR edge suppresses the energy band near Fermi level causing change of properties from metallic to semi-metallic in zigzag-edged GNRs (ZGNRs), which can further be explained as a consequence of the edge polarization effects. Tuning NDR behavior by introducing defects and impurities near the edges of ZGNRs has also been report by Cao and coworkers^55^, and by modifying the passivation of the edge as reported by An and coworkers^56^. In this context, in our present work, we will show that N-doping ZnO nanoribbons can tune the NDR, and cause a suppression of the conduction channel at Fermi level as well. (iii) Quantum coherence effect model, which originally can explain both quantum tunneling and NDR behaviors of Esaki diode by constructive and destructive interferences, respectively. Step-like ZGNR with different step widths have been shown by An and coworkers^57^ to yield NDR behavior. In another related work, An and coworkers also reported^58^ that NDR can be tuned by introducing an extended defect into ZGNRs. They showed that the current of GNR with an upward-triangle defect can be surprisingly larger than the case of pristine GNR due to the defect-induced symmetry breaking and inducing more conductive channels. However, flipping upside down the triangular defect makes the current depressed and yields NDR behavior^58^.

The NDR effect, which features the decreasing current with increasing voltage, has gained wide applications ranging from logical circuits^59^ to memory devices^60^, switches^61–63^, amplifiers^64^, oscillators^34^, and gas sensors^65^. For instance, in the arena of gas-sensing applications, in our recent work^65^, we reported that NDR in ZnO-NR:N can play a major role in inducing high selectivity toward detecting H_2_ molecules. Being a reduced gas, H_2_ molecule not only passivates the dangling bond due to the existence of unpaired electron in the nitrogen impurity, but rather rectifies the IV-characteristics and yields an enormous sensor response. In the present work, we employ the same computational technique, based on ATK-package^51^, to investigate the origins (i.e., search for fundamental reasons for its existence and its mechanisms) of NDR behavior in N-doped ZnO-NRs. These are intrinsic properties that exist in these systems before the occurrence of any gas adsorption and deserve an independent study. The paper is organized as follow: section 2 gives details on the computational method and model; and section 3 shows the results and their discussion. Our main findings are summarized in the last section.

## Results and Discussion

### Transport properties of ZnO-NR based devices

**Figure 1:**
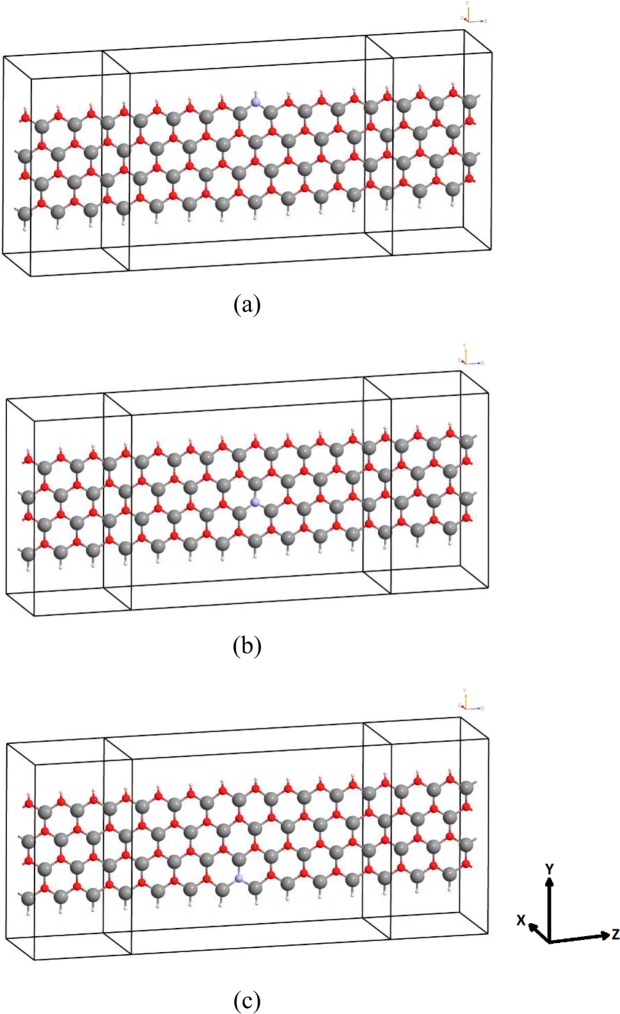
Relaxed structures of ZnO-NR:N devices in which nitrogen dopant is located at (**a**) Upper edge; (**b**) Center; (**c**) Lower edge. Used colors are: O-atoms in red, Zn-atoms in grey, H-atoms in yellow, N-atom in light blue.

Transport properties of N-doped ZnO-NR are systematically investigated in the framework of NEGF. Figure 1 shows the two-probe ZnO-based nano-devices, which consist of three parts: (i) left electrode, (ii) central scattering region, and (iii) right electrode. Each electrode and the central scattering region of device are composed of three and eight channels along the zigzag direction (i.e., z-direction), respectively. The overall length and width of the central scattering region are 27.17 and 11.80 Å, respectively. All the dangling bonds at zigzag edges of all ZnO devices are saturated by hydrogen passivation.

**Figure 2:**
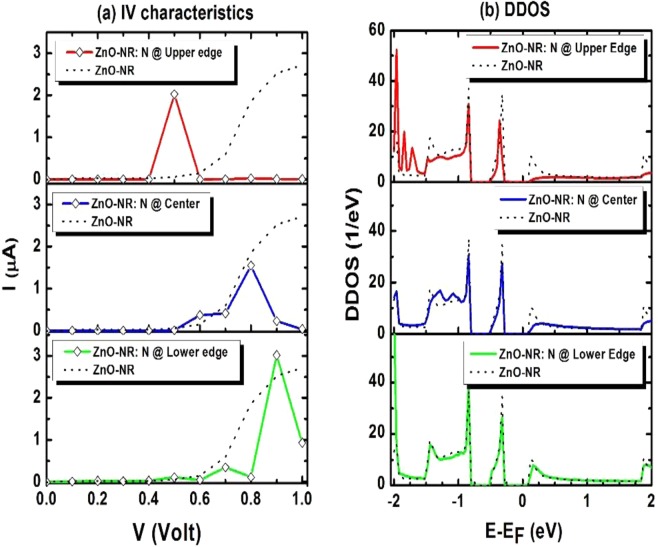
(**a**) IV characteristics and (**b**) Device density of states (DDOS) for the three devices. Fermi energy is taken as an energy reference. The largest Top-to-Valley Current Ratio corresponds to device with N dopant located at Upper Edge (in the oxygen-rich edge).

**Figure 3:**
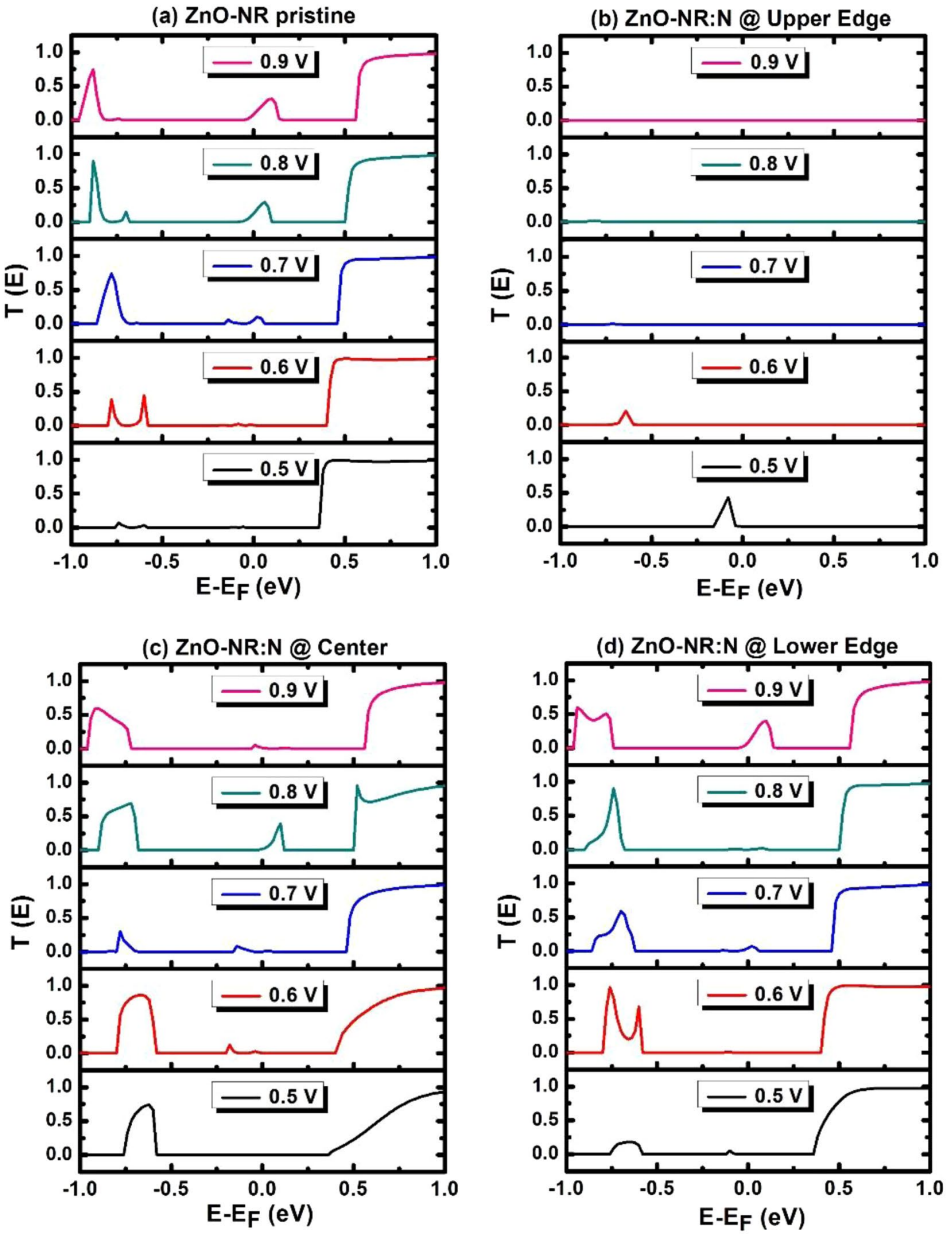
Transmission coefficient versus energy and bias. For devices: (**a**) pristine ZnO-NR, (**b–d**) ZnO-NR:N with N varying its location from O-rich edge to center to Zn-rich edge of NR.

Figure 2 displays the results of IV-curves and device density of states (DDOS) for three device-samples of N-doped ZnO-NR, where N atom is located at: (i) the upper edge of NR (i.e., along oxygen-rich edge) and surrounded by 2 hydrogen atoms and 1 zinc atom; (ii) the center of NR surrounded by 3 zinc neighbors; and (iii) the lower edge of NR (near Zn-rich edge) surrounded by 3 zinc neighbors. Figure 2a displays the IV characteristics of these respective three samples in solid curves, whereas the dotted curve, corresponding to the IV characteristics of pristine ZnO-NR, is shown just as a reference. The three devices with N doping show evidence of NDR behaviors. Figure 2b displaying DDOSs, calculated at zero bias (V = 0) for the same three devices, show that both the device band gap and Fermi level being invariant when the impurity N changes its location (i.e., E_g_ ≈ 0.43 eV remains the same). Perhaps the only residual difference occurs in the filling of the conduction-band edge (i.e., the LUMO state), whose density of states increases as the N get moved toward lower edge of NR. Within the interval [0,1] Volt, Fig. 2a shows the occurrence of maximum current at biases 0.5 V, 0.8 V and 0.9 V in the three devices, respectively. The top-to-valley current ratios (TVCRs) for these devices are: 5549, 6.8, and 3.2, respectively. It seems that as soon as the bias crosses the threshold of bandgap to yield an electric current, some conduction-band channels get suppressed and such suppression does depend on the location of the impurity N-atom with respect to the NR-edges. One may summarize the following scenarios to simultaneously take place: (i) N has five electrons in its outer shell and its substitutional doping to oxygen (which has 6 electrons in its outer shell) would yield an unpaired electron to form like a dangling bond on the N dopant; somehow like a p-type doping (see discussion on the electronic structure below). Nonetheless a compromised effect can take place as the dangling bond needs one electron to become saturated/passivated while this electron makes the site negatively charged and repel the other electrons to come close. (ii) Unlike the atoms at the body of the nanoribbon, the hydrogen atoms at the edges have weak bonds and electrons there do predominantly populate Fermi level. The current density should be very high at the edges of the nanoribbon as they provide very conductive channels with less impedance, especially on the oxygen-rich edge. (iii) Having said these, it seems that moving N atom toward the oxygen edge would cause high impedance and high TVCR, which are attributed to the suppression of many conductive channels as it is illustrated in Fig. 3 (next discussion).

Figure 3 displays the transmission coefficient versus energy for various biases ramping from 0.5 V to 0.9 V for four devices, including (a) pristine ZnO-NR and (c-d) N-doped ZnO-NR with N atom changing position from O-rich edge to center then to Zn-rich edge, respectively. Fermi energy is taken as an energy reference (E_F_ = 0). Even though the bias crosses the bandgap of the scattering zone (i.e., E_g_ ≈ 0.44 eV), vis-à-vis conduction, many bands along the NR seem to be not percolating and just to form suppressed channels. (a) Fig. 3a, corresponding to pristine ZnO-NR, focusing at energy region around Fermi level, a conduction channel starts to develop when the bias reaches V = 0.7 V. Consistently, in Fig. 2a, as shown in dotted curve, the current starts growing at the same bias (0.7 V). (b) Fig. 3b, corresponding to N-doped ZnO-NR with N atom being placed at the upper edge of NR (i.e., along the oxygen-rich edge), near Fermi level, there exists just one conduction channel when a voltage V = 0.5 V is applied. No other conduction channels exist when a bias V in the range 0.5 < V ≤ 1.0 V is applied. Although DDOS of this device, shown in Fig. 2b, shows the evidence of existence of quantum states in this energy interval; yet its seems that those states are quantum-mechanically curdled or confined due to the effect of defect^33,34^ (impurity blocking the conduction states “LUMO”, see below for more details). It is amazing that LUMO states percolated the current at such bias V = 0.5 V then higher CB states seem either having flat band (not conductive channels) or not in resonance with Fermi levels of the two leads. (c) Fig. 3c, corresponding to N-doped ZnO-NR with N atom being placed at the center of NR, focusing on energy region around Fermi level, a conduction channel starts to develop at a bias V = 0.6 V, then it gets closer to E_F_ with larger amplitude when bias V = 0.8 V. Corroborated with this, the current reaches its maximum value at V = 0.8 V in Fig. 2a (central panel). At such bias, one expects good connection between Fermi levels from leads to scattering region meanwhile the ZnO-NR’s band at Fermi level to be dispersive (conducting). (d) Fig. 3d, corresponding to N-doped ZnO-NR with N atom being placed at the lower edge (near Zn-edge), within the energy region around Fermi level, It shows a small peak when V = 0.7 V and further develops into a much larger peak when V = 0.9 V. Consistent with these two peaks, the IV characteristics of this device (shown in lower panel of Fig. 2a), shows accordingly two peaks at V = 0.7 V and 0.9 V, of small and large amplitudes, respectively.

**Figure 4:**
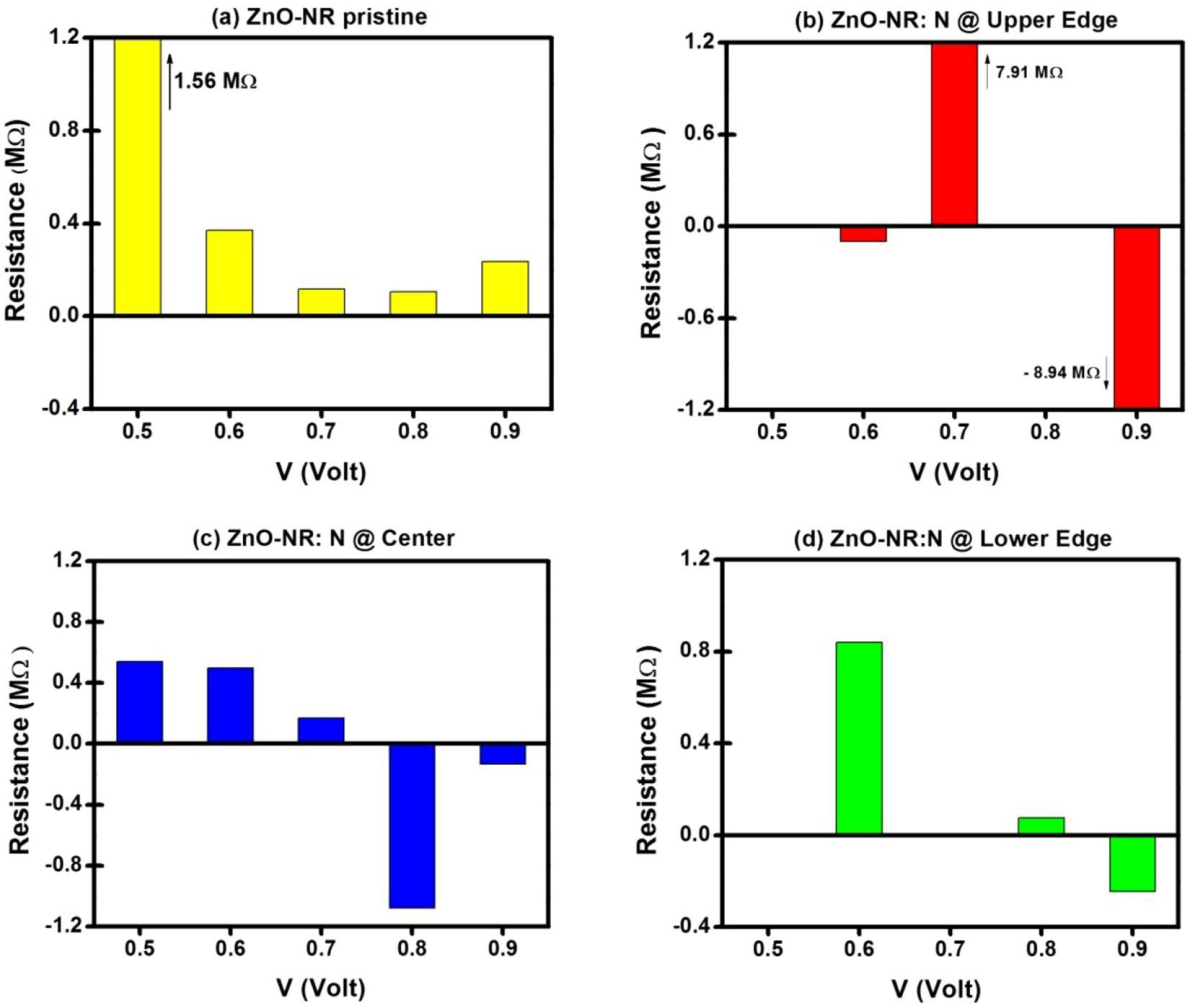
Differential Resistance versus bias for the four devices. Resistance scale is chosen to zoom in at level of magnitude of NDR. The largest NDR correspond to device with N dopant located at Upper Edge (in the oxygen-rich edge).

Figure 4 displays the differential resistance versus bias for the four devices, which were discussed in the previous figure. We recall in this figure that the Mott’s minimum metallic conductivity would yield a maximum measurable resistance of about 0.2 MΩ (see Appendix). So, one should basically supress any resistance of magniture larger than about 1 MΩ, as it would originate from very weak current that can’t be experimentally detected. To our best knowledge, experimental mearurements of resistance in ZnO thin films were reported by Wisz’s group^66^ to be no more than 1 MΩ and by Al-Hardan’s group^67^ to be less than 3 MΩ. One more remarke is about the large existing fluctuations in differential resistance which may be attributed to the universal conductance fluctuations in disordered 1D-2D systems. These fluctuations might be due to the effects of defects in causing severe back-scatterings as well as the ultra-norrowness of the nanoribbon (i.e., with limitation to 4 perculating chanels) which attempts to cause conductance quantization. The compromise of these latter two effects would yield values of conductance not being integers of conductance quanta 2e^2^/h (see Appendix). Moreover, the simultaneous effects (Length of scattering zone being smaller that the coherence length and the NR having 4 perculating chennels, together with defect back-scattering events) can yield large conductance fluctuations. (a) Fig. 4a, corresponding to pristine ZnO-NR, shows the differential resistance calculated for the bias interval 0.5 ≤ V ≤ 0.9 V. The pristine sample is clear of NDR behavior and the differential resistance is always positive. As it is revealed in the IV-characteristics, the differential resistance starts large as soon as current starts flowing in the device (as V crosses E_g_), then reduces to a minimum at a bias of about V = 0.8 then raises again. (b) Fig. 4b, i.e., corresponding to ZnO-NR:N with N atom being placed at upper-edge of NR (i.e., at O-rich edge), shows the highest NDR with an algebraic value of about – 9 MΩ when a bias V = 0.9 V is applied. Such kind of high resistance value is just theoretically displayed (i.e., it cannot be experimentally measured as said earlier) in order to show the effect of N-atom when located in the oxygen-rich edge to block the passage of current. (c) Fig. 4c, corresponding to ZnO-NR:N with N placed at center of NR, has NDR smaller in magnitude than the case of (b), in which N atom is displaced from upper edge toward the center of NR. The minimum value of NDR is about – 1.0 MΩ, which is achieved under bias V = 0.8 V. (d) Fig. 4d, corresponding to ZnO-NR:N with N atom placed at lower edge of NR (i.e., near Zn-rich edge), has the smallest NDR in magnitude, whose algebraic value is of about −0.25 MΩ, to occur when a bias of 0.9 V is applied. Thus, there is a reduction in the magnitude of NDR when the N-dopant is moved from upper to center to lower edge of NR (i.e., from oxygen-rich edge toward zinc-rich edge) revealing that the conduction is predominantly carried out by the oxygen edge in the absence of dopant. The channel gets blocked when the N atom is placed there.

### Electronic properties of ZnO-NRs

**Figure 5:**
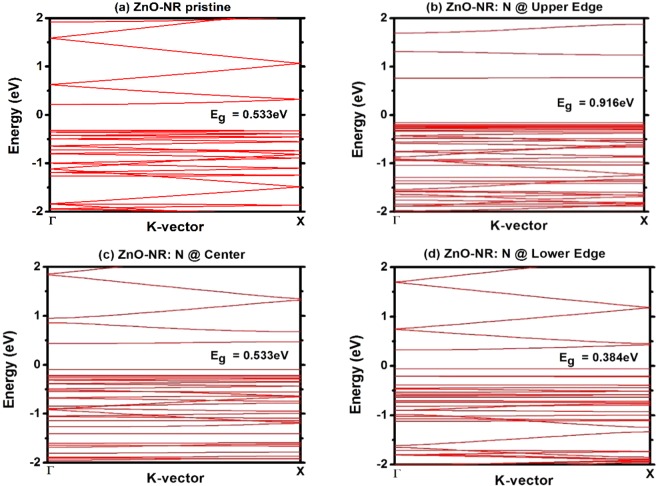
Band structures of 1D-periodic samples corresponding to the scattering regions of the four studied devices. Band-gap energy is largest when N dopant is at O-rich edge. Note also the variations of the VB and CB dispersions.

Using DFT without NEGF methods, one can study pristine and N-doped ZnO-NR samples without the inclusion of electrodes. Such investigation constitutes another perspective or approach in understanding the origins and possible mechanisms causing the NDR in these devices. Four samples, shown in previous figure, have been relaxed and used in the calculations of bands and charge densities. Figure 5 shows the band structures of the four samples, where periodic boundary conditions were applied only along the z-direction. The results show the following: (i) In case of pristine ZnO-NR shown in Fig. 5a, the band-gap energy is Eg = 0.533 eV and Fermi level lays in the middle of the gap as in case of an intrinsic semiconductor. The conduction bands look dispersive. Holes likely have heavier masses than electrons. (ii) In cases of N-doped ZnO-NR shown in Fig. 5b–d, the band-gap energy reduces from E_g_ = 0.916 eV, to 0.533 eV and to 0.384 eV when the N-dopant is moved from upper edge to center then to lower edge of the NR, respectively. (iii) Fermi level lays near the valence band (VB) edge, which reveals that N dopant is playing the role of an acceptor. (iv) Only the lowest conduction-band appears to be flat, as it attributed to a localized state/orbital on a defect. The lowest CB becomes more dispersive as N-atom is moved toward lower edge of NR. (v) Many valence bands appear to be flat and get rather discretized in energy as N-atom is moved toward the lower edge. This discretization of top VBs is attributed to quantum-confinement effect. Apparently, charge get more and more localized around N-dopant when it is moved further downer close to the lower edge (see below for more details in the charge-density plots).

**Figure 6:**
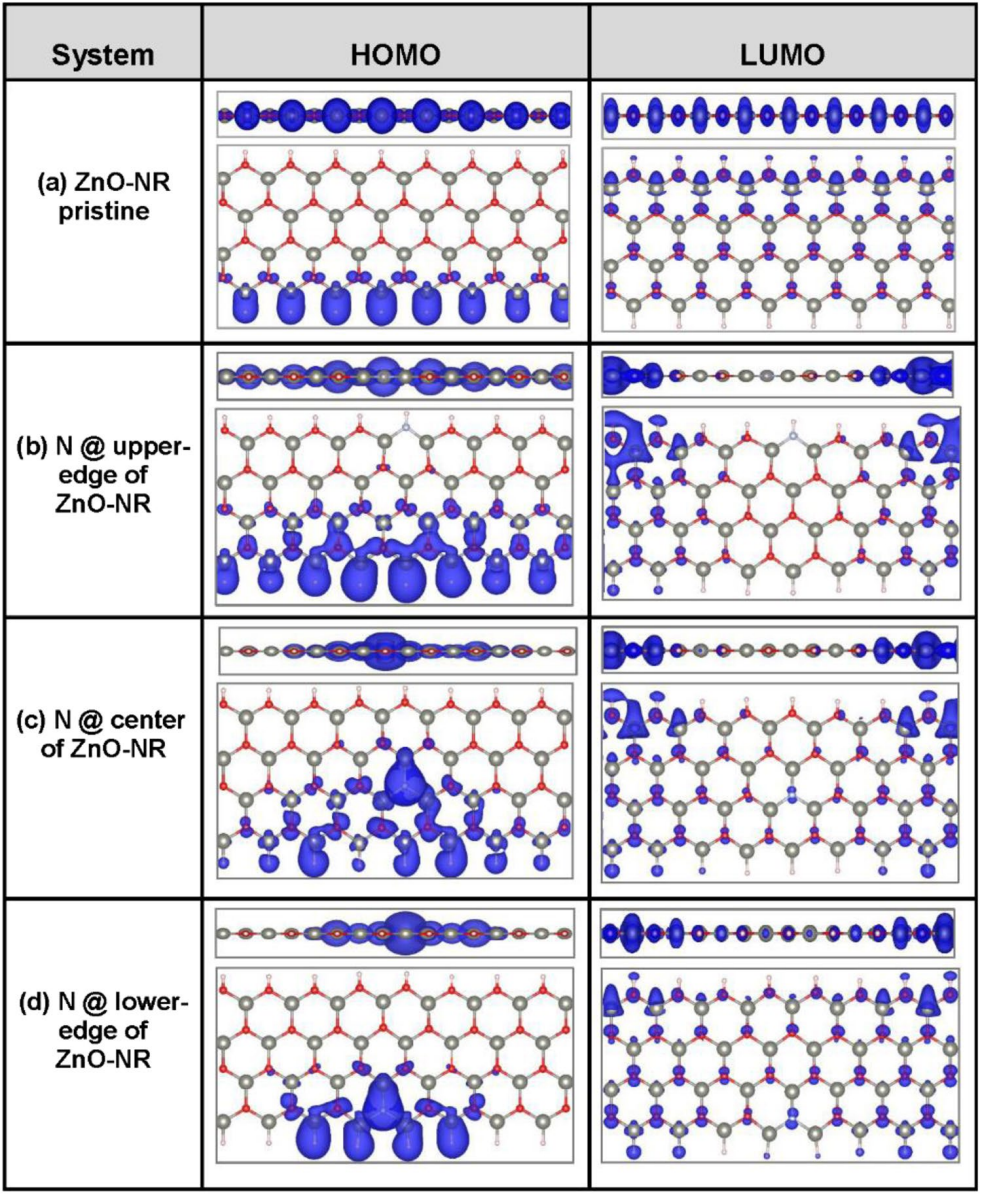
Charge-density plots of the probability-density of HOMO and LUMO eigen-states in 4 samples. (**a**) Pristine ZnO-NR; and (**b**–**d**) correspond to the scattering regions of the three devices, in which N-dopant is varying its location from oxygen-rich edge to center to zinc-rich edge, respectively. Both Side and Top views are shown.

Figure 6 displays charge density plots of probability density of both the highest occupied molecular orbital (HOMO) and the lowest unoccupied molecular orbital (LUMO) for the three samples under focus as well as pristine ZnO-NR, which is taken as a reference. Both side and top views are shown. The probability density is shown in blue color. (a) In case of pristine ZnO-NR, both HOMO and LUMO are Bloch-like states being extended along z-direction. Notably, the HOMO is supported by the Zn sites located at lower edge. Likely, Zn atom has its d-states completely filled and should be deep in energy, laying much below the Fermi level, that can be considered as core states (see, for instance, the first-principle work reported by Topsakal and coworkers^68^). Meanwhile as far as the covalent bonding is concerned, the external s-states of Zn atoms are predominantly contributing to the composition of the valence band. This picture (Fig. 6a HOMO) reveals that the Zn-H bonds are weaker than the O-H bonds and the former do populate the HOMO states just below Fermi level. This is consistent with the bond-dissociation energies reported by Luo^69^: E_b_(Zn-H) = 0.89 ± 0.02 eV/bond being weaker than E_b_(O-H) = 4.461 ± 0.003 eV/bond. On the other hand, the LUMO state should be predominantly populated by O sites, especially those near O-H at the upper edge. Consequently, the conduction is expected to be carried out by these oxygen atoms at the upper edge. (b) In case of ZnO-NR with N dopant located at upper edge, the HOMO remains to be supported by the weakest Zn-H bonds (i.e., by Zn-located at lower edge) but the LUMO gets fragmented on both sides, with respect to z-direction, of the sample as indication of scattering due to defect. This fragmentation is the reason why the lowest CB in Fig. 5b to be flat. (c) In case of ZnO-NR, with N atom being moved to the center, the high electronegativity of N atom starts playing a role so that the HOMO state gets clustered around the N-site. This charge confinement causes the top VB to become discretized and flat, as shown in Fig. 5c. For the LUMO state, the charges still behave like in previous case being fragmented on both sides, along z-direction, of the ZnO-NR. Such fragmentations of LUMO cause the lowest CB to remain flat. (d) In case of ZnO-NR, with N dopant being placed near the lower edge, the confinement of HOMO state at N-dopant’s vicinity becomes even stronger. Consequently, more bands become flat at the top of VB with clear discretization, as shown in Fig. 5d. The LUMO becomes less fragmented than before, as N-dopant moved away from the O-rich edge originally confining the LUMO state. Consequently, kind of a small dispersion in the lowest CB starts to appear, revealing a bit delocalization of LUMO state as is corroborated in Fig. 5d.

## Conclusions

A combination of DFT and NEGF methods is employed to study the origins (i.e., the existence and the mechanisms) of NDR behaviors in N-doped ultra-narrow ZnO-NRs. In testing the position of N-dopant with respect to the edges of NR, three different locations are considered: (i) at the oxygen-rich edge; (ii) at the center; and (iii) at the Zn-rich edge. Our results show the following:

1. In case of pristine ZnO-NR, the current density is found to be predominantly carried out by the O-rich edge, as the states of that region populate Fermi level;
2. In case of ZnO-NR:N, all the three locations of N-dopant yield NDR behaviors. The results of IV-curves show that both magnitude of resistance and TVCR reduce when the N-atom is displaced from O-rich edge to center to Zn-rich edge. It seems that the unpaired electron in N atom is causing the curdling/localization of the wavefunction^70^. This latter behavior, in turn, causes strong back-scattering and the suppression of conductive channels (i.e., NDR).
3. Another remark about the results of IV-curves is that NDR occurs at different energies with the following trend: When N is placed at the O-rich edge, it blocks the current at Fermi level (i.e., E = EF ≈ 0.5 eV). When N is moved to the center, it suppresses the conductive bands at E = 0.8 eV. Last, when N is moved to the Zn-rich edge, it hampers the conduction in channels of energy about E = 0.9 eV.

Thus, after an analysis based on calculations of transmission coefficient versus bias (with V = 0.5–0.9 Volt), band structures, and charge-density plots of HOMO/LUMO states, one is able to draw a conclusion about the origins of NDR. The unpaired electron of N dopant is causing the curdling/localization of wavefunction, which in turn causes strong back-scattering and suppression of conductive channels. These effects manifest themselves in the drawback of electric current (or so called NDR).

### Computational methodology

In this study, all calculations were carried out in the framework of DFT and NEGF using Atomistic Tool Kit (ATK) code^71–73^. Three different locations for the N-dopant in ZnO-NR were systematically investigated: (i) N @ oxygen-rich edge being just within the O-edge line, (ii) N @ center, and (iii) N @ zinc-rich edge being near Zn-edge (see Fig. 1. Double-zeta-polarized basis set of local numerical orbitals was adopted with kinetic energy cutoff of 2040 eV. All atoms in ZnO-NRs were allowed to relax until the Hellmann-Feynman forces on all atoms and the total energy became less than 0.01 eV/Å and 10^-5^ eV, respectively, using DFT within the generalized gradient approximations (GGA) of Perdew-Burke-Ernzerhof (PBE) exchange-correlation functional^74^. Monkhorst-Pack technique was used for the Brillouin zone sampling^75^.

The transport properties (IV-curves) of all devices were calculated by two-terminal Landauer-Büttiker formula^76^:

1 $$I({V}_{b})=\frac{2e}{h}{\int }_{-\infty }^{+\infty }\,T(E,{V}_{b})[{f}_{L}(E-{\mu }_{L})-{f}_{R}(E-{\mu }_{R})]dE$$

where *V*_*b*_, *T(E, V*_*b*_), *μ*_*L/R*_, and *f*_*L/R*_ are the applied forward bias, bias-dependent transmission coefficient, electrochemical potential and Fermi-Dirac distribution function of the left/right electrode, respectively. More details on NEGF-based calculations can be found in the literature^71–73^. Based on IV-curves of ZnO devices, the differential resistance can be estimated as:

2 $$R=\frac{dV}{dI}=\frac{1}{G}$$

where the expression of conductance G within the scheme of Landauer is summarized in Appendix (d). We emphasize that TranSIESTA-package uses Landauer formulation of conductance. Hence, within the scheme of Landauer conductance, the electric current is expressed in terms of transmission probability. The electrodes are usually described as bulk. In case of existence of a defect in the sample, transmission predicts the probability for the electron to tunnel cross the sample and reach a resonant state in the electrode. It seems that scheme of conductivity is not taking care of the variable-range hopping transport. Actually, in our present case, we have just 1 impurity site and IV-curve is calculated to assess the passage of current from left to right electrodes.

## Appendix: Some important tips on quantum transport in mesoscopic systems

- ***Universal conductance fluctuations***:
  The measured electrical conductance in mesoscopic systems usually varies from sample to sample as being controlled by scattering events^77–79^. Within the scheme of the localization theory, the one-parameter scaling theory of Abrahams *et al*.^80^ predicts the absence of quantum diffusion in disordered 1D and 2D systems and that all eigen-states to be exponentially localized. In this 1-parameter scaling theory, the mobility edge exists only in 3D disordered systems to separate between extended and exponentially-localized electronic states. Further progress was done due to the two-parameter scaling theory by Kaveh^81^ on 2D finite-size disordered systems. Using the Anderson model, Kaveh predicted the existence of a pseudo-mobility edge in 2D to separate two types of localized states; the power-law localized states towards the center of the band and the exponentially-localized states towards the tails of the band. Yet, concerning the origins of the universal conductance fluctuations (UCF), the claims attributed them to both disorder (potential ensemble) and coherence effects of electronic wavefunctions (phase ensemble)^82^. These two effects can appear simultaneously in the dc-conductivity when the system size is smaller than both coherence length (L_Φ_) and localization length (ξ), as Fermi level lies on defect states. Furthermore, the conductance fluctuations are even more pronounced when electrical transport is taking place in the weak localization regime (such as our devices). In weakly-localized systems, the fluctuation in conductance could reach the fundamental conductance “quanta”, ${G}_{o}=\frac{2{e}^{2}}{h}=7.75\times {10}^{-5}{\Omega }^{-1}$, where e is the electron charge and h is Planck constant, regardless of the number of conducting channels.
  In disordered systems, where scattering events are predominant, a self-averaging quantity, like DOS, obeys the central-limit theorem and thus the iterations over the realization-random sets converge fast. Unlike this, the dc-conductivity is non-self-converging quantity and requires many iterations over random sets of phases. In brief, in nano-devices such as our present case, the magnitude of the universal conductance fluctuation is mainly influenced by two factors, the symmetry and the size of the sample. A third factor been recently added is due to the anisotropy of Fermi surface^83^.
- ***Minimum Metallic Conductivity*** **:**
  In dirty (disordered) metals, it has been noticed that the dc-conductivity vanishes as temperature goes to 0 K in continuous way within a minimum value of ${\sigma }_{min}\cong 0.025{e}^{2}/\hslash a$ (i.e., ${G}_{min}\cong 6.08\times {10}^{-6}{\Omega }^{-1}$, which corresponds to a maximum resistance ${R}_{max}\cong 0.2\,M\Omega ),$ where a is the average distance between impurities. This was reported by Mott^84^ and experimentally observed by Davis and Compton^85^. From Drude model of conductivity, minimum metallic conductivity can easily be understood as the mean free-path cannot be smaller than the lattice spacing^86^. Until present, experimental evidence continues to appear and corroborate the existence of minimum metallic conductivity in various systems. For instance, recently, Farka and coworkers reported that conduction in the elastic scattering regime in conductive polymers of PEDOT:Sulphate to display a minimum metallic conductivity at low temperature of 4 K^87^.
- ***Conductance quantization:***
  The conductance quantum, denoted as ${G}_{o}=\frac{2{e}^{2}}{h}$, can be achieved in clean quantum point-contact system limit^88^. The fabrication of quantized-conductance atomic switch (QCAS) was achieved, by Terabe group^89^, in which the current exhibited stair-way behavior with conductance quantized into multiples of G_o_. Such characteristics being achieved at room temperature and under ambient conditions would pave the way for these switches to be used in logical switches in future computers. Furthermore, quantized-conductance majorana of zero-mode – a type of localized quasiparticle – was recently fabricated for the same purpose of applications in quantum computing^90^. Nonetheless, one should make it clear that the phenomenon of quantization of conductance should not mean that the conductance of any system must be an integer multiple of G_o_, as that can be reached only in case of realization of conductive channel(s) operating within the ballistic regime (i.e., with a transmission probability of unity). In case of disorder, it is usual that the transmission probability to be less than one, then the conductance would be even less than G_o_ (i.e., G < G_o_). The total conductance of any system is, of course, the superposition of those due to its percolating channels.
- ***Landauer conductance:***
  The problem of electronic transport is a non-equilibrium one. The traditional method, known as linear response theory (LRT), considers the system at equilibrium and treats the driving electric force using a first-order perturbation approximation (i.e., the scheme of Kubo-Greenwood formalism of conductivity^91,92^). In 1970, Landauer^93^ proposed a new formalism of conductance, which is probe-dependent and in which the system is composed of a sample as interacting with the two probes. The transport through the scattering zone of sample is treated by a transmission coefficient T(E). The conductance (G) of the system in assumption of having one channel under a small applied bias (V) is given by:
  3 $$G=\frac{I}{V}=\frac{2{e}^{2}}{h}T({E}_{F})$$
  where *e* is the electron charge, *E*_*F*_ is Fermi energy, and *h* is the Planck constant.

In case of existence of multi-channels within a cross-sectional area of a quasi-1D sample, then the Landauer formula can be generalized to the form:

4 $$G=\frac{2{e}^{2}}{h}\sum _{i,j}\,{T}_{ij}({E}_{F})$$

where *T*_*ij*_ is the probability that a carried (say electron) transmits from the i-th mode at the left to j-th mode at the right side of the sample; and *E*_*F*_ is the Fermi energy. In case of small bias, the general expression of electric current would be:

5 $$I={\int }_{0}^{\infty }\frac{dE}{e}[f(E+eV)-f(E)]G(E)$$

The Hamiltonian of the system is written as *H* = *H*_0_ + *U*, where *H*_0_ is the Hamiltonian of the sample as uncoupled to the electrodes, and *U* represents the coupling between the sample and the electrodes. The transmission operator becomes:

6 $$T(E)=U+UG(E)U$$

where $G(E)=\frac{1}{E-H+i\eta }$ is the Green’s function.

The transmission probability in equation (4) is related to the matrix elements of T(E) operator in equation (6) by the following relation:

7 $$\sum _{i,j}\,{T}_{ij}(E)=4{\pi }^{2}\sum _{l,r}\,{|{T}_{lr}|}^{2}\delta (E-{E}_{l})\delta (E-{E}_{r})$$

where δ is the Dirac delta function, E_l_ and E_r_ are the energy modes at left and right electrodes, respectively. Under bias *V*_*b*_, they are related as: ${E}_{l}={E}_{r}-e{V}_{b}$, so the current flows from left to right electrodes.
